# The 2018 Nobel Prize in medicine goes to cancer immunotherapy (editorial for BMC cancer)

**DOI:** 10.1186/s12885-018-5020-3

**Published:** 2018-11-12

**Authors:** Zong Sheng Guo

**Affiliations:** 1 UPMC Hillman Cancer Center, Pittsburgh, PA 15213 USA; 2 University of Pittsburgh School of Medicine, Pittsburgh, PA 15213 USA

The 2018 Nobel Prize in Physiology or Medicine goes to James P. Allison and Tasuku Honjo “for their discovery of cancer therapy by inhibition of negative immune regulation”.

For many decades, the standard treatments for cancer have been mostly surgery, chemotherapy and/or radiation therapy. Despite these treatments, most patients with advanced malignant disease had miserable survival rates. New thinking and novel strategies for cancer therapy were badly needed. Cancer immunotherapy seemed to be the answer. There is a long history of efforts to take advantage of a patient’s own immune system as a therapeutic modality for cancer. The earliest effort may trace back to the observation of William B. Coley, who as a surgeon correlated the occurrence of postoperative infection with improved clinical outcomes in cancer patients in 1893 [1]. Later on, several modalities of cancer immunotherapies were approved, including bacillus Calmette-Guerin, interferon-α, and interleukin-2 (IL-2). The work with IL-2 in treating cancer substantiated the important role of adaptive immunity in cancer therapy.

Through decades of hard work, we started to gain an understanding of the basic mechanisms of T cell activation. Naïve T cells acquire two key signals from antigen presenting cells (APC), with the first one being presentation of the antigen-derived epitope peptides in the context of MHC class antigens to the T-cell receptor on T cells. The second signal is mainly through the interaction of co-stimulatory molecules, such as CD28, expressed on most T cells with its natural ligands CD80 and CD86 on APC. These co-stimulatory signals, or so called T-cell “accelerators”, are required to trigger a full-blown immune response against cancer or infectious diseases. Nevertheless, although we had accumulated quite a bit of knowledge about antitumor immunity prior to 2010, clinical studies of cancer immunotherapies had been mostly disappointing. Various cancer vaccines had generated poor clinical responses in thousands of cancer patients, and so did adoptive transfer of tumor-infiltrating lymphocytes (TILs) [2]. During the mid-1990s, a lot of investigators had doubts about the future of cancer immunotherapy.

## A new concept of regulated immunity and new principle for immunotherapy

One key intrinsic property of the immune system is to maintain a delicate homeostasis, in order to get rid of pathogens, yet not elicit autoimmune diseases. Therefore, in the 1990s, investigators hypothesized that “brakes” as well as “accelerators” exist for the activated T cells.

Allison studied the T-cell protein CTLA-4 in the 1990s. He and other teams of scientists had made the observation that CTLA-4 functions as a brake on T cell activities. He had developed an antibody that could block CTLA-4 function. He set out to investigate the hypothesis that CTLA-4 blockade could release the T-cell brake to allow the immune system to attack cancer cells. Mice with cancer had been cured by treatment with the anti-CTLA-4 antibody thanks to unlocked antitumor T-cell activity [3, 4]. He continued intense efforts to develop the strategy into a cancer therapy regimen for human. In 2010, a pivotal clinical study showed striking effects in patients with advanced melanoma.

Honjo discovered PD-1 expressed on the surface of T-cells in 1992 [5]. It took many years of work to unravel its role in immune response. The results showed that PD-1 operates by a different mechanism from CTLA-4. In preclinical studies, Honjo and others showed that PD-1 blockade was a promising strategy in the fight against cancer [6, 7]. In 2012, a key clinical study demonstrated obvious efficacy of anti-PD-1 antibody in cancer patients. This treatment led to long-term remission and a possible cure for multiple patients with metastatic cancer.

The work done by Allison, Honjo, together with many others, have opened up a new avenue for cancer immunotherapy, which led to approval by the authorities for the use of immune checkpoint blockade to treat at least ten types of cancers so far, and many more to come.

## The Nobel Prize work opens up a bright future for cancer immunotherapy

The Prize winning work launched a new field that now goes far beyond CTLA-4 and PD-1. At least three areas of ongoing research may lead us to novel fundamental knowledge of immunology and brand new regimens of immunotherapy for cancer and other immunological diseases. First, other co-inhibitory signaling pathways, such as HVEM-BTLA and Galectin-9-TIM3, are being studied in cancer and other diseases [8]. Once we know more about these inhibitory signaling pathways, we may design rational combinational strategies to concurrently target two or more inhibitory pathways to gain better therapeutic efficacy. Secondly, one rational strategy would be to combine immune checkpoint blockade with other immunotherapy regimens or other standard of care regimens in order to eliminate primary cancer and metastases more effectively. One such strategy has been to combine anti-PD-1/PD-L1 or anti-CTL4 with oncolytic viruses (OVs). This combinatorial strategy deems to be rational as OVs induce inflammation in cancer, which in turn increase TILs, upregulate immune checkpoint molecules such as PD-1/PD-L1, and as a result, transforms a “cold” tumor to a “hot” one immunologically [9, 10]. As we have suggested in our recent study, this combinatorial therapy may be applicable to a much wider population of cancer patients than the monotherapies do. So far, data from the first two clinical trials demonstrated this combination of OV with immune checkpoint antibody achieved much higher efficacy with shown toxicity in patients with advanced melanoma [11, 12]. Finally, other types of cancer immunotherapies, including adoptive transfer of CAR T cells [13], TCR-modified T cells [14], and cancer vaccines using neo-antigens [15, 16], have made significant progress in recent years, and has shown promise in clinics. It is envisioned that some selective rational combinations of these anti-cancer agents with immune checkpoint blockade will work more effectively on some cancer patients as personalized medicine.

In summary, after this major recognition of the importance of immunity, investigators from this field are highly excited and will spare no efforts to make this regimen of cancer therapeutics more efficacious. The future of this therapy looks brighter.
